# A novel frameshift variant in *MSH2* (p.Q170Rfs4) associated with suspected Lynch syndrome in a Chinese family

**DOI:** 10.3389/fmed.2026.1774595

**Published:** 2026-03-31

**Authors:** Xiawen Yang, Yan Huang, Shan Xiao, Xiufang Wang, Haichun Ni, Chengzhi He, Aiping Deng, Jing Fu, Lijun Xiong, Juyi Li

**Affiliations:** 1 Department of Pharmacy, The Central Hospital of Wuhan, Tongji Medical College, Huazhong University of Science and Technology, Wuhan, Hubei, China; 2 Department of Geriatric Outpatient, The Central Hospital of Wuhan, Tongji Medical College, Huazhong University of Science and Technology, Wuhan, Hubei, China; 3 Department of Pain, The Central Hospital of Wuhan, Tongji Medical College, Huazhong University of Science and Technology, Wuhan, Hubei, China; 4 Department of Pathology, The Central Hospital of Wuhan, Tongji Medical College, Huazhong University of Science and Technology, Wuhan, Hubei, China; 5 Department of Gastrointestinal Surgery, The Central Hospital of Wuhan, Tongji Medical College, Huazhong University of Science and Technology, Wuhan, China; 6 Department of Physical Examination, The Central Hospital of Wuhan, Tongji Medical College, Huazhong University of Science and Technology, Wuhan, Hubei, China

**Keywords:** genetic counseling, Lynch syndrome, mismatch repair, *MSH2* variant, whole-exome sequencing

## Abstract

**Objective:**

This study aimed to characterize the pathogenic variants in three colon cancer families suspected of Lynch syndrome (LS), providing experimental evidence for precision screening and genetic counseling of the disease.

**Methods:**

Three suspected LS families were first identified, and subsequently, immunohistochemical analysis was performed on colon tissue samples from probands to assess the expression of four DNA mismatch repair proteins. Whole-exome sequencing was conducted to screen for potential pathogenic variants within the families. The SWISS-MODEL online platform was used to predict the three-dimensional structures of the mutant and wild-type proteins based on bioinformatics analysis. The predicted structures were then visualized using PyMOL software.

**Results:**

Two known *MSH2* missense variants were identified: NM_000251.3:c.2633A>T:p.E878V in Family 1 and NM_000251.3:c.998G>A:p.C333Y in Family 2. A novel variant, designated as NM_000251.3:c.507del:p.Q170Rfs*4, was identified in the *MSH2* gene of Family 3. This variant is caused by the deletion of an adenine at nucleotide position 507 within the *MSH2* coding sequence, resulting in a frameshift. Consequently, glutamine at amino acid position 170 is altered to arginine, and a premature termination codon is introduced three residues downstream. This frameshift is predicted to generate a truncated protein of only 172 amino acids.

**Conclusion:**

The *MSH2* missense variant NM_000251.3:c.2633A>T:p.E878V was classified as a variant of uncertain significance regarding its role in LS. In contrast, the NM_000251.3:c.998G>A:p.C333Y missense variant was confirmed as pathogenic. Furthermore, the novel frameshift deletion NM_000251.3:c.507del:p.Q170Rfs*4 was also identified as a pathogenic variant.

## 1 Introduction

Colorectal cancer (CRC) has a lifetime individual risk of 50–70% and ranks as the third most common cancer globally (1, 2). Epidemiological data from Europe indicate a significantly higher incidence in males compared to females, with approximately 3.5–4.2% and 2.4–3.2% of new cases occurring in males and females, respectively. Furthermore, although mortality rates are lower in developed countries, the number of CRC cases in these regions is five times that in developing countries. In developing regions, the ratio of mortality to detected cases remains notably high (3). In China, CRC is the second most common malignancy overall, with an age-standardized incidence rate (ASIR) of 2.3 per 10,000. It ranks third in both sexes, with ASIRs of 2.81 and 1.94 per 10,000 for males and females, respectively (4).

Lynch syndrome (LS) is an autosomal dominant hereditary cancer syndrome and represents the most common cause of inherited CRC (5). The occurrence of LS is closely related to pathogenic germline variants (PGVs) of four key genes (*MLH1, MSH2, MSH6*, and *PMS2*) in the DNA mismatch repair (MMR) system (6, 7). Dysfunction in any MMR allele among the four genes can lead to deficiency in the MMR system, thereby significantly increasing the risk of multi-organ carcinogenesis in carriers (8). In individuals with LS, both CRC and endometrial cancer exhibit notable early-onset characteristics (9). Beyond the cancers mentioned above, individuals with LS also exhibit an elevated risk of tumors in other sites, including the ovaries, small intestine, stomach, pancreatobiliary tract, brain, upper urinary tract, and skin (10).

Therefore, it is crucial to characterize the mutation characteristics of LS in the Chinese population, and to provide further genetic counseling based on the results of genetic testing. In this study, we performed comprehensive clinical and genetic evaluations in three families with suspected Lynch syndrome, including colonoscopy examinations, histopathological analysis with hematoxylin and eosin (HE) staining, immunohistochemistry (IHC), Whole-Exome Sequencing (WES), bioinformatic predictions using multiple in silico tools, Sanger sequencing, and three-dimensional structural predictions. Genetic sequencing of three clinically suspected LS families revealed the presence of three *MSH2* variants, including two known missense variants (NM_000251.3:c.2633A>T:p.E878V and NM_000251.3:c.998G>A:p.C333Y) in addition to a novel frameshift deletion (NM_000251.3:c.507del:p.Q170Rfs*4). This study aimed to characterize their clinical significance to inform genetic counseling and personalized management for affected carriers.

## 2 Methods and materials

### 2.1 Baseline characteristics of participants

Three families were recruited in this study. Proband I was a 42-year-old male admitted due to epigastric pain persisting for 6 months. His mother had a history of colon cancer. Proband II was a 59-year-old female admitted with paroxysmal distending pain in the epigastric and right upper abdominal regions. Her father died of rectal cancer, and her mother died of pancreatic cancer. Proband III was a 45-year-old male admitted for intermittent abdominal pain over 2 days. His mother was diagnosed with both colon cancer and uterine cancer. Each of the three probands included in the cohort was evaluated clinically and deemed to be a suspected case of LS. Prior to study initiation, written informed consent was voluntarily by every participant to confirm their willingness to participate. Additionally, this research protocol was formally reviewed and granted approval by the Ethics Committee of Wuhan Central Hospital.

### 2.2 Colonoscopy examination

All patients underwent colonoscopy following standard bowel preparation. Examinations were performed by experienced gastroenterologists using a high-definition colonoscope (Olympus Corporation, Tokyo, Japan). The entire colonic mucosa was carefully inspected, and representative images of abnormal findings were captured for documentation.

### 2.3 Histopathological and immunohistochemical staining

Tumor specimens underwent formalin fixation, paraffin embedding, and sectioning, followed by Hematoxylin–Eosin (HE) staining to evaluate histopathological morphology. To assess MMR deficiency, IHC was performed to detect the protein expression of MLH1, MSH2, MSH6, and PMS2. Tissue sections were labeled with corresponding antibodies, and loss of nuclear staining was used to determine the functional status of the MMR proteins.

### 2.4 Genomic DNA extraction and whole-exome sequencing

Genomic DNA was extracted from tumor tissues of the probands. Exonic regions were enriched using the Agilent SureSelect Human All Exon V7 kit. The resulting libraries were subjected to paired-end sequencing (2 × 150 bp) on an Illumina NovaSeq 6,000 platform. The reference genome used for WES analysis was UCSC hg19 (NCBI build GRCh37) (11, 12).

### 2.5 Bioinformatic analysis

To predict the functional impact of the identified variants, multiple in silico bioinformatics tools were utilized. These included SIFT, PolyPhen-2, MutationTaster, LRT, FATHMM, and REVEL. Population frequencies were assessed using the 1,000 Genomes Project, the NHLBI Exome Sequencing Project (ESP6500)and the Genome Aggregation Database (gnomAD).

### 2.6 Sanger sequencing

Sanger sequencing was performed to validate candidate variants using genomic DNA extracted from the peripheral blood of each proband. Primer sequences used for amplification were as follows: Proband I, Forward Primer: (5′ → 3′): TGCTGTCTTCTCTCATCGTGTCC; Reverse Primer (5′ → 3′): GATCATGCAGATGTATATACTCGAC-3′. Proband II, forward primer: (5′ → 3′): GTGGTTTTGCTGGGGAGAA; Reverse Primer (5′ → 3′): ATCATCGGGTAACTCGAGTTAC. Proband III, forward primer: (5′ → 3′): ACTTAGGCTTCTCCTGGCAATCTC; Reverse Primer (5′ → 3′): CTTTCTAGGGCTGGAATCTCCT. The resulting PCR amplicons were purified and sequenced on an ABI 3500 DNA Analyzer.

### 2.7 Three-dimensional structure

The predicted tertiary structure of the MSH2 protein was obtained using the I-TASSER software1, and the molecular structure was visualized using the PyMOL software2.

## 3 Result

### 3.1 Clinical features

**Figure 1:**
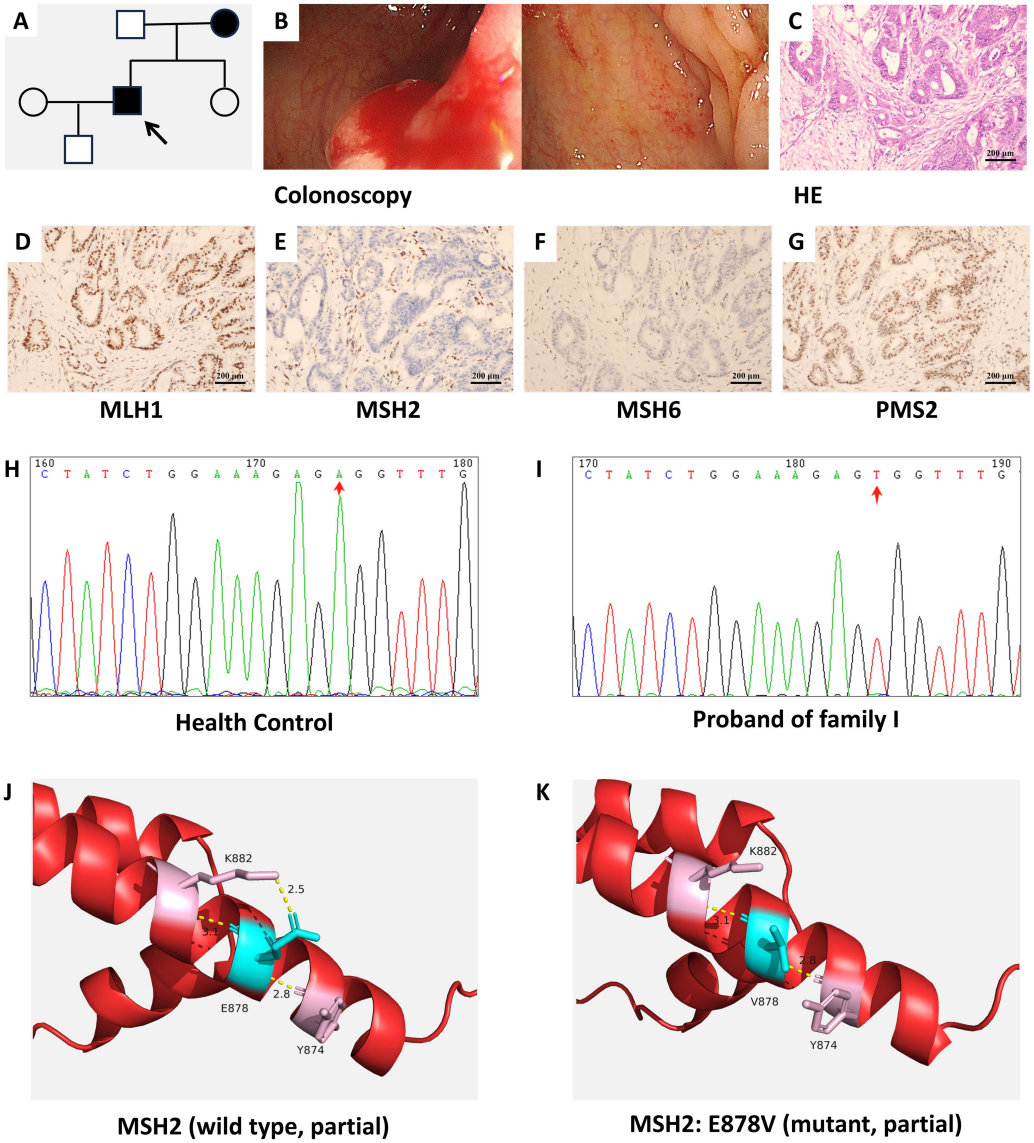
**(A)** Pedigree of family I with suspected LS. The proband I is indicated by an arrow. Filled symbols denote individuals affected with LS or other LS-associated malignancies (circles: females; squares: males). **(B)** Colonoscopy findings of proband I, showing a smooth mucosal surface with clearly visible vascular networks and increased bleeding tendency. **(C)** HE staining of the colonic tissue from proband I revealed a moderately differentiated ulcerative adenocarcinoma. **(D–G)** IHC staining results for MLH1 **(D)**, MSH2 **(E)**, MSH6 **(F)**, and PMS2 **(G)** in proband I. **(H,I)** Sequencing analysis identifies a *MSH2* variant (*MSH2*: NM_000251.3:c.2633A>T:p.E878V) in proband I. **(J,K)** Predicted three-dimensional local structures of the wild-type MSH2 protein **(J)** and the *MSH2*: E878V variant **(K)**.

**Figure 2:**
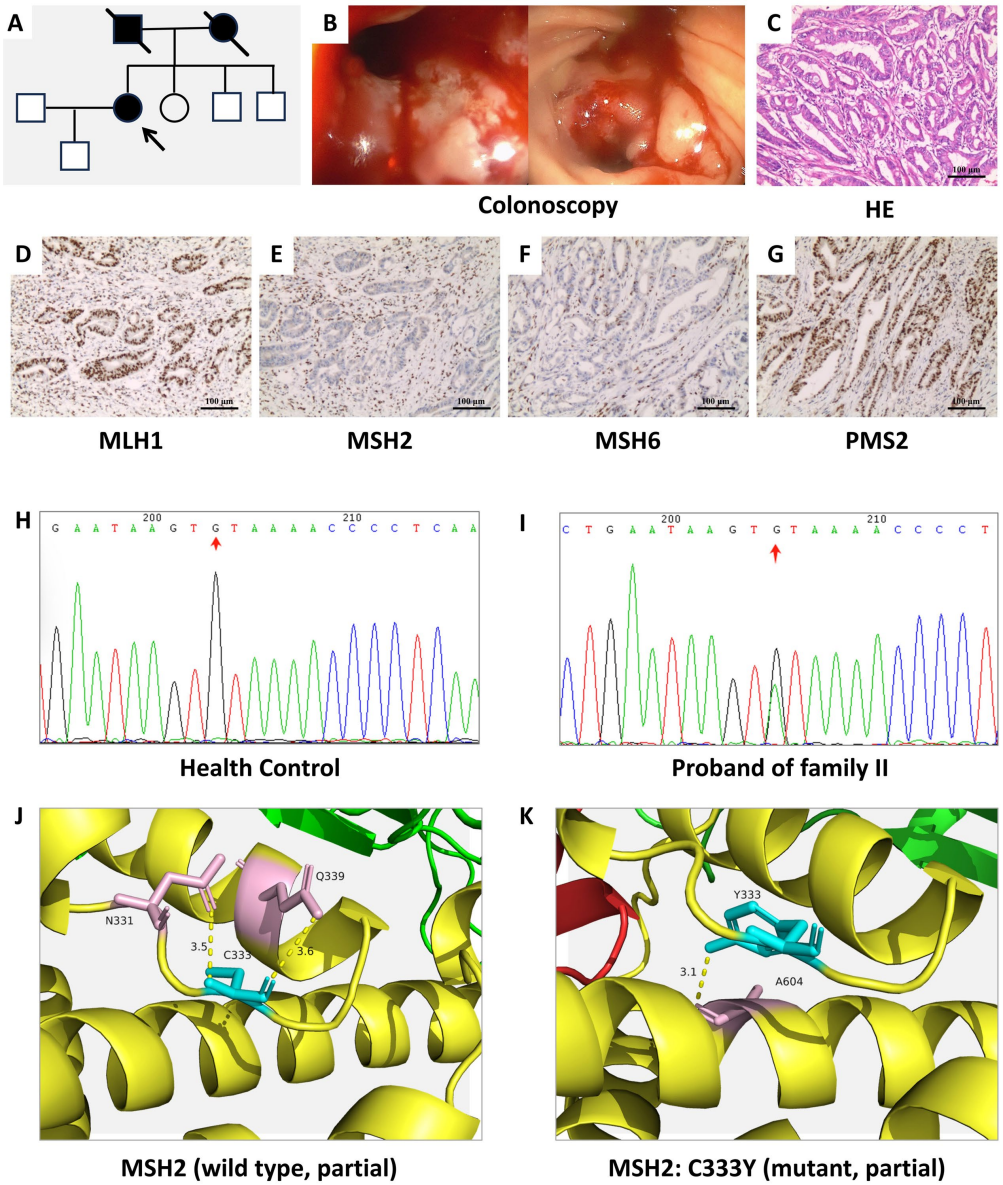
**(A)** Pedigree of family II with suspected LS. The proband II is indicated by an arrow. Filled symbols denote individuals affected with LS or other LS-associated malignancies (circles: females; squares: males). **(B)** Colonoscopy findings of proband II, revealing ulcerated colonic mucosa with a friable texture and a tendency to bleed easily. **(C)** HE staining of the colonic tissue from proband II demonstrated a moderately to poorly differentiated ulcerative adenocarcinoma. **(D–G)** IHC staining results of MLH1 **(D)**, MSH2 **(E)**, MSH6 **(F)**, and PMS2 **(G)** in proband II. **(H,I)** Sequencing analysis identifies a *MSH2* variant (*MSH2*: NM-000251.3: c.998G>A: p.C333Y) in proband II. **(J,K)** Predicted three-dimensional local structures of the wild-type MSH2 protein **(J)** and the *MSH2*: C333Y variant **(K)**.

**Figure 3:**
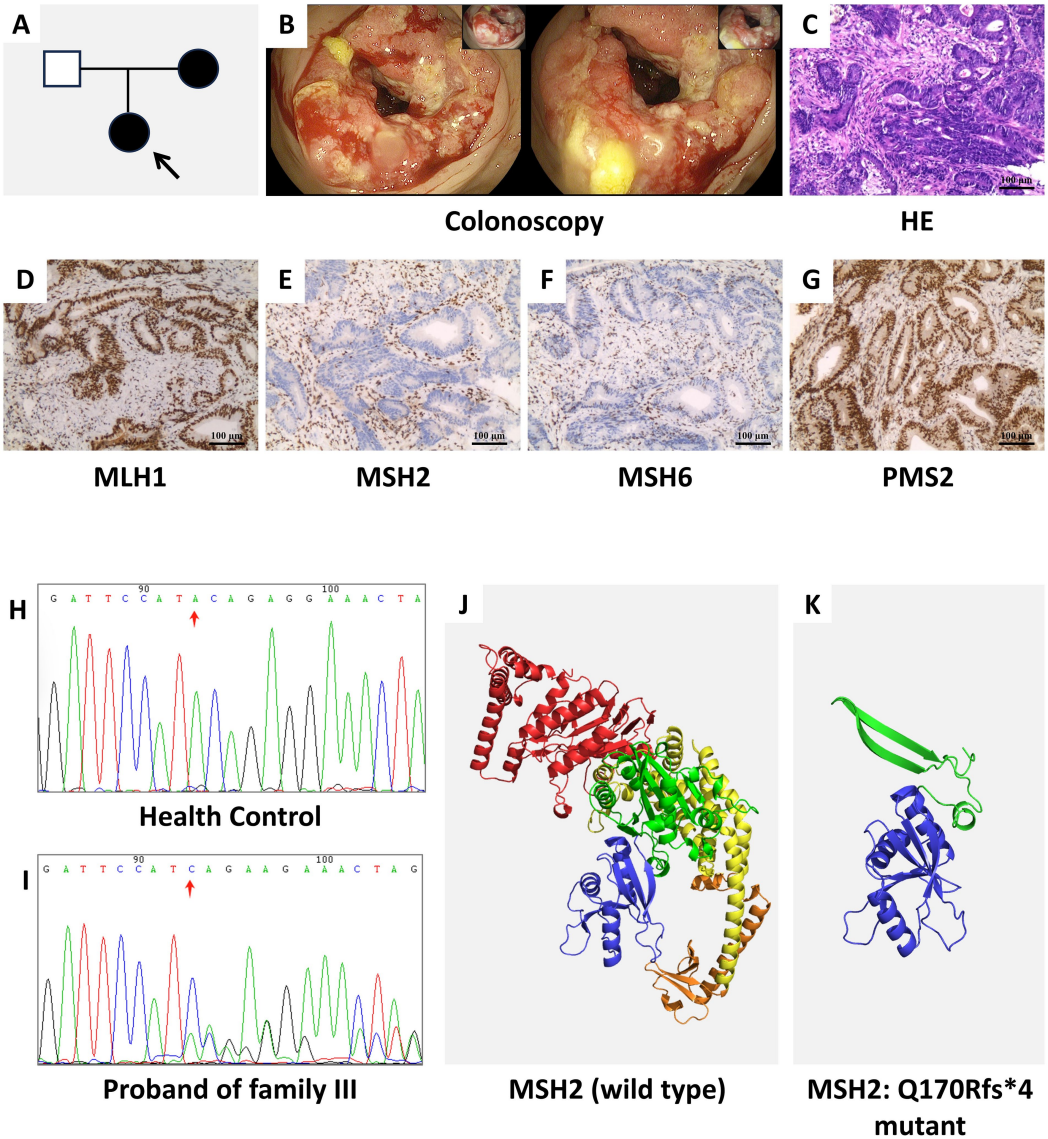
**(A)** Pedigree of family III with suspected LS. The proband III is indicated by an arrow. Filled symbols denote individuals affected with LS or other LS-associated malignancies (circles: females; squares: males). **(B)** Colonoscopy findings of proband III, showing congested and edematous colonic mucosa that is friable and prone to bleeding. **(C)** HE staining of colon tissue from proband III, revealing a moderately to well-differentiated ulcerative adenocarcinoma. **(D–G)** IHC staining results of MLH1 **(D)**, MSH2 **(E)**, MSH6 **(F)**, and PMS2 **(G)** in proband III. **(H,I)** Sanger sequencing analysis identified a novel *MSH2* variant (*MSH2*: NM_000251.3:c.507del:p.Q170Rfs4) in proband III. **(J,K)** Predicted three-dimensional structures of the wild-type MSH2 protein **(J)** and the *MSH2*:Q170Rfs4 variant **(K)**.

The pedigrees of Families I, II, and III are shown in Figures 1A, 2A, 3A, respectively. Colonoscopy of proband I (Figure 1B) showed a smooth mucosa with clearly visible vascular networks, although the mucosa appeared fragile. In proband II (Figure 2B), colonoscopy revealed extensive ulceration of the colonic mucosa, which was friable and exhibited spontaneous bleeding. For proband III (Figure 3B), colonoscopic evaluation demonstrated circumferential mucosal erythema and edema, with marked friability and spontaneous hemorrhage.

### 3.2 Histopathological and immunohistochemical findings

HE staining of the colon tissue (Figure 1C) indicated a moderately differentiated ulcerative adenocarcinoma, with focal areas exhibiting features of mucinous adenocarcinoma. IHC staining results revealed robust nuclear expression (positive staining) of MLH1 (Figure 1D) and PMS2 (Figure 1G) in the tumor specimens, while MSH2 (Figure 1E) and MSH6 (Figure 1F) were negative. HE staining (Figure 2C) revealed an ulcerating adenocarcinoma of moderate-to-poor differentiation. IHC staining results demonstrated robust nuclear expression of MLH1 (Figure 2D) and PMS2 (Figure 2G), while MSH2 (Figure 2E) expression was absent and MSH6 (Figure 2F) expression was partially absent. Figure 3A delineates the pedigree of family 3. Colonoscopic evaluation of proband III (Figure 3B) revealed circumferential mucosal erythema and edema, with marked friability and spontaneous bleeding. HE staining (Figure 3C) demonstrated an ulcerating adenocarcinoma of moderate-to-high differentiation. IHC staining results showed intact nuclear expression of MLH1 (Figure 3D) and PMS2 (Figure 3G), whereas MSH2 (Figure 3E) and MSH6 (Figure 3F) were not expressed.

### 3.3 Genetic and bioinformatic analysis

Table 1 summarizes the WES data obtained from probands I, II, and III. We analyzed pathogenic genes associated with LS, such as *MLH1, MSH2, MSH6*, and *PMS2*, as well as several less common genes (*EPCAM*) (13). The analysis results confirmed that *MSH2* gene was the pathogenic gene in all probands. Specifically, in proband I, the variant was located at chromosome 2 position 47,708,009 and designated as MSH2: NM_000251.3:c.2633A>T:p.E878V. Functional assessment using multiple bioinformatics prediction tools yielded the following scores: SIFT (0.008), Polyphen2_HVAR (0.782), Polyphen2_HDIV (0.998), MutationTaster (1), LRT (0.000), FATHMM (−2.89), and REVEL (0.757). These results suggest that the variant is likely pathogenic. However, due to the currently limited supporting evidence, its clinical significance remains uncertain. In proband II, the variant was identified at chromosome 2 position 47,643,490 and designated as *MSH2*: NM_000251.3:c.998G>A:p.C333Y. Consistent predictions from multiple bioinformatics tools (SIFT: 0.008; Polyphen2_HVAR: 0.996; Polyphen2_HDIV: 1.0; MutationTaster: 1; LRT: 0.000; FATHMM: -3.34; REVEL: 0.951) indicated that this variant is pathogenic and functionally damaging. Combined with clinical evidence, we classify this variant as pathogenic. In proband III, the variant was identified as *MSH2*: NM_000251.3:c.507del:p.Q170Rfs*4. Searches in public population genomic databases, including the 1,000 Genomes Project (1,000 g), ESP6500, and gnomAD, revealed no records of this variant. Therefore, it was determined to be a novel, previously unreported variant.

**Table 1:** Whole-exome sequencing detail of the patient.

Exome capture statistics	Proband I	Proband II	Proband III
Total (bp)	59,016,008 (100%)	49,743,686 (100%)	82,618,912 (100%)
Duplicate (bp)	17,239,769 (29.21%)	12,330,156 (24.79%)	22,313,531 (27.01%)
Mapped (bp)	58,971,487 (99.92%)	49,705,628 (99.92%)	82,571,988 (99.94%)
Properly mapped (bp)	58,501,336 (99.13%)	48,395,304 (97.29%)	82,175,296 (99.46%)
PE mapped (bp)	58,939,734 (99.87%)	49,679,200 (99.87%)	82,536,298 (99.90%)
SE mapped (bp)	63,506 (0.11%)	52,856 (0.11%)	71,380 (0.09%)
With mate mapped to a different chr	293,892 (0.50%)	982,794 (1.98%)	220,488 (0.27%)
With mate mapped to a different chr ((mapQ> = 5))	242,010 (0.41%)	911,804 (1.83%)	182,154 (0.22%)
Initial bases on target (bp)	60,456,963	60,456,963	60,456,963
Initial bases on or near target (bp)	136,297,444	136,297,444	136,297,444
Total effective yield (Mb)	8,748.92	7,367.01	12,312.34
Effective yield on target (Mb)	6,596.23	5,098.10	9,024.46
Fraction of effective bases on target (%)	75.40%	69.20%	73.30%
Fraction of effective bases on or near target (%)	91.40%	90.30%	91.40%
Average sequencing depth on target	109.11	84.33	149.27
Bases covered on target (bp)	59,933,296	60,036,841	60,233,373
Coverage of target region (%)	99.10%	99.30%	99.60%
Fraction of target covered with at least 100× (%)	38.10%	32.20%	56.30%
Fraction of target covered with at least 50× (%)	63.30%	66.30%	80.30%
Fraction of target covered with at least 20× (%)	85.70%	89.40%	93.90%
Fraction of target covered with at least 10× (%)	93.40%	95.50%	97.40%
Fraction of target covered with at least 4× (%)	97.50%	98.30%	99.00%
Total SNPs	85,949	99,155	104,412
Novel SNPs	1965	1,021	1,549
Total InDels	9,843	10,708	12,568
Novel InDels	1835	1,087	2035
Gender	Male	Female	Male

PE, paired-end sequencing; SE, single-end sequencing; bp, base pairs; Mb, megabase pairs; chr, chromosome; mapQ, mapping quality; SNPs, single nucleotide polymorphisms; InDels, insertions and deletions.

### 3.4 Sanger sequencing results

Sanger sequencing identified three *MSH2* variants in the probands. Proband I carried a rare variant (NM_000251.3:c.2633A>T:p.E878V) (Figures 1H,I). Proband II harboured another variant (MSH2: NM_000251.3:c.998G>A:p.C333Y), resulting from a GG>GA dinucleotide change at position 754 (Figures 2H,I). A novel frameshift variant (NM_000251.3: c.507del, p.Gln170Argfs*4) was detected in proband III; this single-base deletion is expected to cause a frameshift and premature termination (Figures 3H,I).

### 3.5 Protein structure prediction

Protein structure modeling was performed based on the identified variants. The three-dimensional local spatial structure of the MSH2 protein in proband I is presented as follows: the wild-type structure in Figure 1J and the mutant structure (*MSH2*: E878V) in Figure 1K. The results showed that compared with the wild type (14, 15), the c.2633A>T variant replaced polar glutamic acid with non-polar valine at residue 878, altering key hydrogen bonding interactions. In the wild-type structure, E878 forms hydrogen bonds with Y874 (2.8 Å) and K882 (2.5 Å and 3.1 Å), whereas V878 retains only a single bond with Y874 (2.8 Å) and a weakened interaction with K882 (3.1 Å). In proband II, the p.C333Y variant (Figure 2J, wild-type; Figure 2K, mutant) replaces a cysteine with a tyrosine at residue 333. While wild-type C333 forms hydrogen bonds with N331 (3.5 Å) and Q339 (3.6 Å), mutant Y333 establishes only one hydrogen bond with A604 (3.1 Å), which is predicted to destabilize the adjacent *β*-strand. In proband III, the p.Q170Rfs*4 variant (Figure 3J, wild-type; Figure 3K, mutant) is a frameshift mutation resulting from a single-base deletion (c.507del). This leads to substitution of glutamine with arginine at codon 170 and introduces a premature termination codon four residues downstream, producing a truncated protein of 172 amino acids. The MSH2 protein has five structural domains (16), this variant results in the loss of Domains 3–5 and alters Domain 2, severely compromising protein integrity.

## 4 Discussion

In recent years, the incidence of CRC has shown a significant upward trend globally. The clinical manifestations of this disease lack specificity, and some cases may even present with no obvious symptoms in the early stages, making early diagnosis crucial for improving patient prognosis. Numerous studies have confirmed that genetic susceptibility contributes significantly to the development of CRC. Detection of specific gene mutations provides key evidence for etiological diagnosis and molecular subtyping of the disease.

In this study, genetic testing was conducted on three families with suspected LS, identifying three clinically significant variant sites: a novel frameshift deletion (NM_000251.3:c.507del:p.Q170Rfs*4), which was not documented in multiple public genetic databases, including gnomAD, a variant of uncertain significance (NM_000251.3:c.2633A>T:p.E878V), and a confirmed pathogenic missense variant (NM_000251.3:c.998G>A:p.C333Y). Both of which have been reported as rare variants, however, the *MSH2* variant (NM_000251.3:c.507del:p.Q170Rfs*4) was defined as pathogenic (PVS1 + PM2 + PM6 + PP4).

Traditional methods for identifying Lynch syndrome patients, such as the Amsterdam II criteria and the revised Bethesda guidelines, rely predominantly on family cancer history (17). However, in China, due to the family planning policy of the past few decades, small families have become dominant nationwide, limiting the availability of complete family lines for clinical evaluation. As shown in this study, incomplete family tree information is a common limiting factor that undermines its reliability as an independent diagnostic criterion. Therefore, combining family history with molecular genetic testing provides a more robust strategy for the diagnosis of LS (18). The proteins encoded by *MLH1* and *MSH2* act as obligatory partners in all MMR heterodimers, explaining why these two genes confer the greatest risk for LS (19). This is reflected in the substantially elevated lifetime CRC risks for *MLH1* (42%) and *MSH2* (33%) pathogenic variant carriers, compared to the lower risks associated with *MSH6* (18%) or *PMS2* (7%) variants (20). In this study, all identified variants in the three suspected LS families were localized within the *MSH2* gene. In proband I, IHC staining analysis of MMR proteins revealed loss of expression for both MSH2 and MSH6. Genetic sequencing identified a rare missense variant, *MSH2*: NM_000251.3:c.2633A>T:p.E878V (rs1573579250). Its DNA sequence has a GAG variant to GTG, resulting in the replacement of the 878th amino acid encoded by glutamic acid (Glu) with valine (Val). Bioinformatics predictions suggest this amino acid substitution may be pathogenic; however, the ClinVar database currently classifies its clinical significance as “uncertain significance” and its specific pathogenic mechanism requires further in-depth investigation.

Proband II harboured the canonical pathogenic allele *MSH2*: NM_000251.3:c.998G>A; p.C333Y (rs63750828).^1^ The HI score for this variant shows sufficient evidence for dosage pathogenicity, while there is currently no evidence related to TS score. This variant leads to the substitution of cysteine to tyrosine at codon 333, breaking the conserved disulfide bridge, destabilizing the MMR heterodimer, and accelerating its degradation via the proteasome (21). According to variant classification criteria, this variant is classified as a Class 5 (pathogenic) variant. In clinical practice, carriers should be closely monitored following high-risk disease management guidelines, and predictive genetic testing is recommended for their relatives (22).

Missense variants account for 20–30% of LS-associated gene alterations, and interpreting their clinical significance is particularly complex. The functional impact of such variants on protein activity varies widely, ranging from mild functional alterations to severe functional defects. Moreover, most missense variants are rare in the general population, and the lack of prior research evidence supporting their pathogenic classification further complicates their interpretation (23). In fact, among the missense variants associated with LS recorded in the ClinVar database, more than one-third remain unclassified and are still designated as variants of uncertain significance (VUS) (24). This adds considerable difficulty for clinicians and genetic counselors in making disease management decisions and assessing cancer risk for patients.

In proband III, a novel variant, *MSH2*: NM_000251.3:c.507del:p.Q170Rfs*4, was identified. Which leads to the loss of the structural domain of MSH2 protein. The absence of several key domains likely disrupts the stability and integrity of the MSH2-MSH6 complex, impairing its functional activity and ultimately leading to defective DNA MMR (25). Therefore, for patients carrying this pathogenic variant, regular health surveillance, including gastrointestinal endoscopy and gynecological endoscopic examinations, should be recommended to enable early diagnosis and precise management of the disease.

In the United States, current clinical guidelines recommend that patients with LS undergo colonoscopic surveillance (every 1–2 years from age 20–25) (27) and consider chemoprevention, a strategy supported by the CAPP2 trial demonstrating that long-term daily aspirin (600 mg for 2–4 years) reduces colorectal cancer (CRC) risk (28). However, prospective data from the CAPP2 cohort indicate that the chemopreventive benefit of aspirin against CRC is confined to carriers of *MLH1* pathogenic variants; no protective effect was observed among *MSH2* pathogenic variant carriers. Beyond pharmacological intervention, lifestyle modification-specifically increased physical activity and reduced adiposity-have also been shown to attenuate LS-associated cancer risk, with a more pronounced protective effect in male carriers (19, 29). Although chemotherapy serves as the primary therapeutic modality for most CRC patients, adjuvant 5-fluorouracil monotherapy tends to confer no significant benefits on patients with stage II and III CRC (30). Immunotherapy-based approaches, such as immune checkpoint inhibitors and vaccination, hold promise as cancer prevention strategies for LS patients, with potential protective effects spanning multiple cancer types (31). PD-1 inhibitors (e.g., pembrolizumab or nivolumab) achieve an objective response rate of 30–40% and a disease control rate of up to 70% in the treatment of refractory CRC (32). The results from a phase I/IIa clinical trial indicated that all 16 CRC patients developed specific humoral and cellular immune responses after completing four vaccine doses, with no significant adverse events reported (33). With the continuous emergence of novel cancer prevention technologies, the importance of efficient and early screening for LS genetic susceptibility is increasingly underscored. Liquid biopsy, as a minimally invasive and highly sensitive detection technique, demonstrates promising application prospects in the screening of LS-associated cancers and warrants further in-depth investigation (34).

One of the main limitations of this study is the lack of co-segregation analysis of the detected variants within families. Future research should prioritize genetic testing of available family members. This will not only provide crucial evidence for the pathogenicity of these variants, particularly variants of uncertain significance, but also allow for a more accurate assessment of their penetrance, thereby optimizing risk management strategies for carriers. Despite the limitations mentioned above, this study underscores the practical value of systematic genetic screening in families that meet relevant clinical criteria. All probands and their relatives with confirmed pathogenic variants received individualized counseling for LS in a clinical setting or via teleconsultation. The counseling covered the inheritance pattern of the syndrome, cancer risks for both carriers and their family members, and available risk-based surveillance strategies, such as regular colonoscopies, endometrial biopsies, and imaging studies. The identification of well-characterized pathogenic variants enables targeted predictive genetic testing of at-risk relatives. This allows for evidence-based active surveillance and preventive interventions in carriers, while non-carriers can be spared unnecessary screening, reflecting the core principle of precision preventive medicine.

In summary, we delineated one novel variant (*MSH2*: NM_000251.3:c.507del;p.Q170Rfs*4) and comfirmed the clinical significance of two rare missense alleles (*MSH2*: NM_000251.3:c.2633A>T:p.E878V and *MSH2*: NM_000251.3:c.998G>A:p.C333Y). These findings expand the Chinese mutational landscape of DNA MMR genes and refine the continuously evolving LS pathogenicity repository, underscoring the pivotal role of MMR testing for diagnosis, risk stratification, and family counseling. On the basis of these data and current evidence, cancer families with documented or suspected MMR deficiency should be offered systematic characterization as a clinical priority. Carriers warrant individualized surveillance integrating endoscopic and imaging modalities for early tumor detection, while therapeutic algorithms should be tailored to the specific variant, gene, and patient phenotype, ultimately improving outcomes and curtailing inter-generational transmission of LS-related malignancies.

## Data Availability

The dataset provided in this study can be found in an online repository. The name and login number of the repository can be found below: NCBI SRA: PRJNA1414680.

## Footnotes

1
https://www.ncbi.nlm.nih.gov/clinvar/variation/91273/
